# Inherited pulmonary cylindromas: extending the phenotype of *CYLD* mutation carriers†

**DOI:** 10.1111/bjd.16573

**Published:** 2018-05-29

**Authors:** S.M. Brown, M. Arefi, R. Stones, P.S. Loo, S. Barnard, C. Bloxham, N. Stefanos, J.A.A. Langtry, S. Worthy, E. Calonje, A. Husain, N. Rajan

**Affiliations:** ^1^ Department of Dermatology Royal Victoria Infirmary Newcastle upon Tyne U.K.; ^2^ Institute of Genetic Medicine University of Newcastle upon Tyne U.K.; ^3^ Department of Pathology Royal Victoria Infirmary Newcastle upon Tyne U.K.; ^4^ Department of Cardiothoracics Freeman Hospital Newcastle upon Tyne U.K.; ^5^ Department of Radiology Royal Victoria Infirmary Newcastle upon Tyne U.K.; ^6^ Dermatopathology Department St John's Institute of Dermatology St Thomas' Hospital London U.K.

## Abstract

**Background:**

Germline mutations in the tumour suppressor gene *CYLD* are recognized to be associated with the development of multiple cutaneous cylindromas. We encountered such a patient who presented with breathlessness because of multiple pulmonary cylindromas.

**Objectives:**

To search for clinical and radiological features of multiple pulmonary cylindromas in a cohort of 16 patients with *CYLD* mutations.

**Methods:**

A retrospective case‐note review was carried out in a tertiary dermatogenetics clinic where *CYLD* mutation carriers are reviewed on an annual basis. In‐depth investigation was carried out for patients with pulmonary tumours.

**Results:**

Four patients had radiological imaging of their lungs, of which two had multiple pulmonary cylindromas that were confirmed histologically. Serial computed tomography monitoring allowed for pre‐emptive endobronchial laser ablation, preventing major airway obstruction and pulmonary collapse.

**Conclusions:**

Pulmonary cylindromas are an unrecognized, but infrequently symptomatic, aspect of the phenotype in these patients that can have implications for patient care. They should be considered in patients with a high tumour burden that present with respiratory symptoms, and where appropriate, monitored with serial imaging.

## Abstract 2

**What's already known about this topic?**

- Germline mutations in the tumour suppressor gene *CYLD* are recognized to be associated with the development of multiple cutaneous cylindromas, spiradenomas and trichoepitheliomas.
- Beyond the skin, salivary gland tumours are seen in less than approximately 5% of patients, but otherwise the tropism of this disease is recognized as exclusively cutaneous.

**What does this study add?**

- We report multiple pulmonary cylindromas in two patients out of a series of 16 patients, a previously unreported aspect of this genetic disease.
- Deep phenotyping with radiological, histological and genetic approaches provides evidence that supports that these tumours are similar to cutaneous cylindromas and share a cytokeratin signature seen in cutaneous cylindroma.

**What is the translational message?**

- Patients with this rare disease should be investigated if they present with new symptoms of respiratory disease.
- These data inform the clinical surveillance and management of patients with this rare condition and also inform the longstanding debate on benign metastases.

https://doi.org/10.1111/bjd.17046 available online

https://goo.gl/Uqv3dl

CYLD cutaneous syndrome (CCS; synonym Brooke‐Spiegler syndrome – OMIM 605041) is an inherited tumour syndrome arising because of germline mutations in the tumour suppressor gene *CYLD*.^1^ Defined by the rare appendageal tumour cylindroma, patients develop numerous cutaneous tumours, and a minority develop a type of salivary gland tumour, membranous basal cell adenoma, which shares similar histology. We encountered a patient in our cohort of well‐characterized *CYLD* mutation carriers who presented with breathlessness and were surprised to discover multiple pulmonary cylindromas. This finding prompted a case review of our cohort of 16 patients, where we found a second individual who had four pulmonary cylindromas. As this rare disease is poorly understood, we carried out deep phenotyping and molecular analyses on the pulmonary cylindromas seen in the two patients with CCS. We carried out detailed comparative histological analyses of skin and lung tumours, including expression of the proliferation marker Ki‐67 and tropomyosin receptor kinase (TRK) B and C, proteins previously reported to be overexpressed in cutaneous cylindroma.^2^ These data extend the phenotype of this genodermatosis, provide guidance on the management of these patients and informs the debate on benign metastases.

## Materials and methods

### Patients

Retrospective review of the case notes and radiological data of 16 patients with CCS that were receiving follow‐up between 1 July 2013 and 1 July 2017 was performed including the index case. Skin and lung samples were obtained from patients with signed consent. Research ethics committee approval was obtained for this work (Hartlepool Research Ethics Committee, REC Ref: 06/Q1001/59; 14/NE/1080).

### Immunohistochemistry

Immunohistochemical staining for Ki‐67, TRKB and TRKC was carried out as described previously.^2^ Tissue sections were dewaxed and rehydrated, following which antigen retrieval was performed in citrate buffer (pH 6·0) for 12 min in a pressure cooker. Sections were then blocked and then probed overnight at 4 °C with primary antibodies against relevant targets.

Secondary HRP (horseradish peroxidase) antibodies were applied the following day and visualized with DAB as per the manufacturer's instructions (Dako‐K4009 Envision, Carpinteria, CA, U.S.A.). Antibodies were obtained from Cell Signaling Technology, Beverly, MA, U.S.A.

### Transcriptomic profiling

RNA was extracted from all samples using the Qiagen Allprep kit (Qiagen, Manchester, U.K.), quantified using the Qubit BR assay (Invitrogen, ThermoFisher Scientific, Paisley, U.K.) and quality assessed using the Bioanalyser (Agilent, Santa Clara, CA, U.S.A.). DNA extracted as part of this protocol was used for Miseq sequencing as described below.

Stranded preparation was performed using the Illumina stranded mRNA kit (Illumina, San Diego, CA, U.S.A.). Libraries were prepared and sequenced using an Illumina Hiseq 2500, giving 45 million paired end reads per sample, which were 100 base pair (bp) in length. FASTQ files were aligned using the splice aware aligner program STAR to generate alignment files.^3^ The read counts for each sample file were counted using the R package Subread.^4^ Differential gene expression analysis was carried out using the package DeSeq2.^5^

### Miseq *CYLD* sequencing

DNA from the same samples were quantified using the Qubit HS assay (Invitrogen). Amplicons were generated that covered the entire locus of *CYLD*, normalized for size, and 1 ng input DNA was used to generate indexed libraries using the Nextera XT protocol (Illumina). Libraries were sequenced using a Miseq v3 flowcell capable of generating 75 bp paired end reads on a Miseq sequencer (Illumina). Alignment of all reads against the hg19 reference genome was carried out and variant calling was performed using Miseq reporter software. Variants were visualized using Integrative Genomics Viewer (Broad Institute, Cambridge, MA, U.S.A.) and coverage was greater than ×2500.

## Results

The characteristics of the patients studied are shown in Table 1. Of the 16 patients reviewed, respiratory symptoms were present only in two. A further two patients had undergone computed tomography (CT) imaging for staging following the diagnoses of poorly differentiated cutaneous cylindrocarcinoma and adnexal carcinoma; CT investigations in both revealed normal lung fields. Three of these four patients carried a germline mutation in *CYLD* (c.2460delC) and were part of a seven‐generation family that has been previously reported (Fig. S1; see Supporting Information),^6^ and the fourth patient with normal lung fields had a distinct mutation (c.1112C>A).

**Table 1:** Cohort of patients with *CYLD* cutaneous syndrome reviewed in this study

Patient	Age at examination	Sex	Genetic mutation	Respiratory symptoms at time of review	Pulmonary imaging
1	80	Female	c.2460delC	Breathlessness	Cylindromas
2	63	Female	c.2460delC	Cough and breathlessness	Cylindromas
3	60	Female	NMD	None	None performed
4	74	Female	c.2460delC	None	None performed
5	47	Female	c.2460delC	None	None performed
6	51	Female	NMD	None	None performed
7	74	Female	c.2460delC	None	None performed
8	74	Female	c.2460delC	None	None performed
9	46	Female	c.2460delC	None	Normal lung fields
10	43	Female	c.2460delC	None	None performed
11	70	Male	c.2460delC	None	None performed
12	53	Female	c.2460delC	None	None performed
13	48	Female	c.2806C>T	None	None performed
14	44	Female	c.2806C>T	None	None performed
15	57	Female	c.1112C>A	None	Normal lung fields
16	37	Male	c.1112C>A	None	None performed

**Figure 1:**
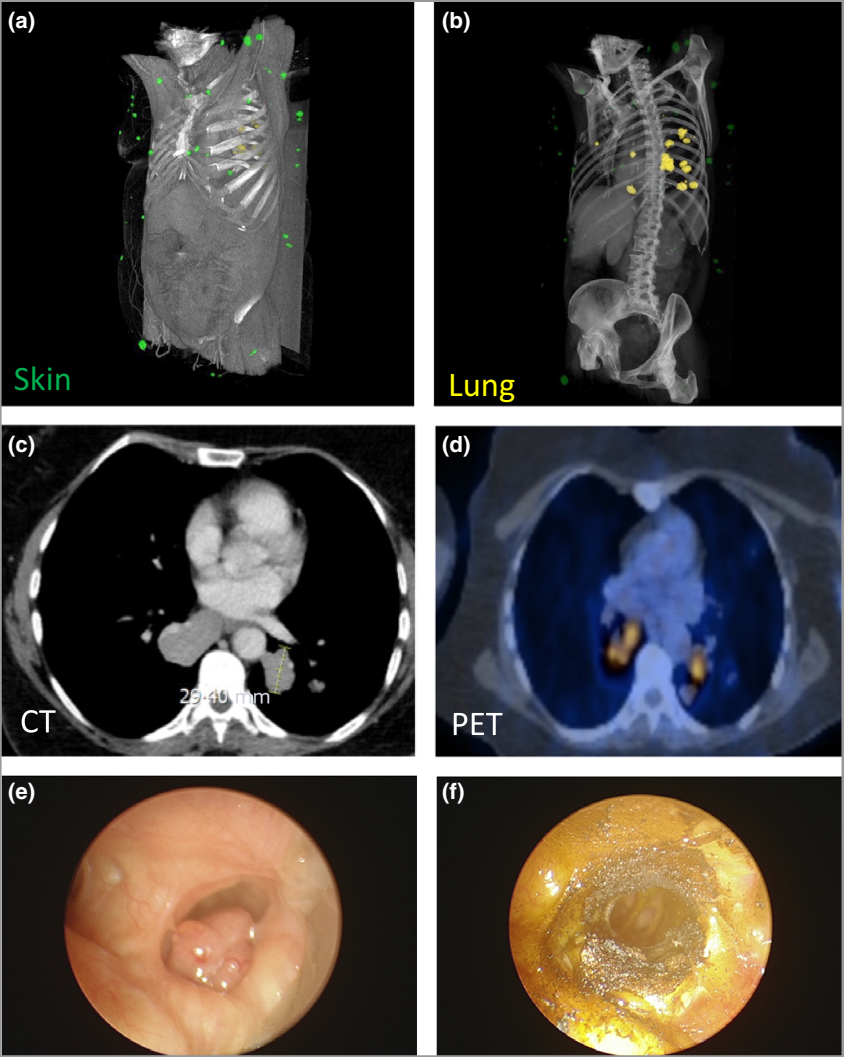
Clinical characterization of pulmonary cylindromas. (a) Three‐dimensional reconstruction and visualization of high‐resolution computed tomography (CT) images demonstrate the presence and morphology of cutaneous cylindromas (green lesions) and (b) pulmonary cylindromas (yellow lesions). (c) High‐resolution CT image of pulmonary cylindroma measuring 29·4 mm. (d) Fludeoxyglucose (FDG) positron emission tomography (PET) demonstrating high uptake in pulmonary cylindromas. Endobronchial views of pulmonary cylindroma (e) before and (f) after Nd‐YAG (neodymium‐doped yttrium aluminum garnet) laser ablation to restore airway patency.

The 64‐year‐old female proband presented with increased breathlessness on exertion and a 2‐year history of cough. She had a severe phenotype, having undergone complete scalp excision at the age of 52, and had numerous cutaneous tumours on her torso, but no history of malignancy. She had a 20‐year history of smoking. Chest radiograph of the proband revealed multiple opacities throughout both lungs. High‐resolution CT imaging demonstrated multiple, lobulated, soft tissue lesions, some of which encroached into large airways (Fig. 1a, b). Ultrasound scan of the salivary glands confirmed absence of a primary salivary gland tumour. The largest tumour in the right lung hilum measured 3·7 cm across and there was no mediastinal or hilar lymphadenopathy noted (Fig. 1c). No hepatic or bony metastases were seen.

**Figure 2:**
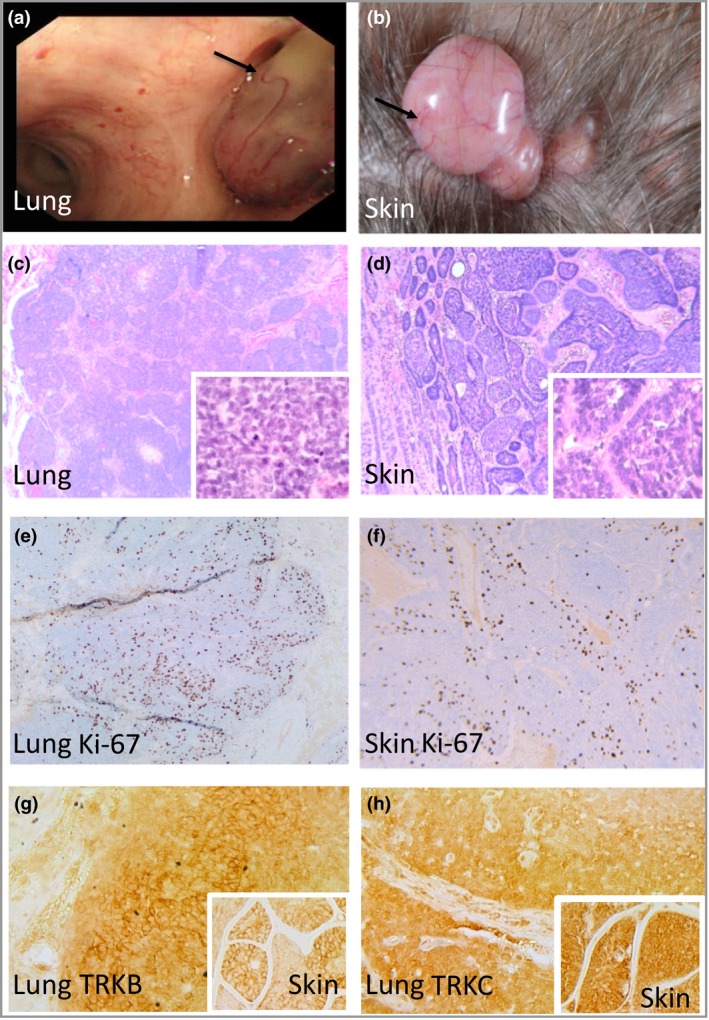
Clinical and histological comparison of cutaneous and pulmonary cylindromas in the proband. (a) Arborizing blood vessels are seen both on pulmonary tumours indicated with black arrow and (b) in cutaneous cylindroma. Histology of (c) pulmonary and (d) cutaneous cylindroma from the same patient showing similarity in morphology (inset – ×40 magnification). Ki‐67 expression in (e) pulmonary cylindroma and (f) cutaneous cylindroma (×10 magnification). (g) Tropomyosin receptor kinase (TRK)B and (h) TRKC expression in pulmonary cylindroma (insets cutaneous cylindroma for comparison; ×40 magnification).

Video‐assisted thoracoscopy demonstrated yellow nodules with arborizing blood vessels, reminiscent of cutaneous cylindromas (Fig. 2a, b). Positron emission tomography demonstrated multiple pulmonary lesions with high fludeoxyglucose (FDG) uptake and one cutaneous lesion on the lower right abdomen with moderate FDG uptake (Fig. 1d). There was no hilar or mediastinal lymph node FDG uptake noted and no hepatic FDG uptake. Serial CT scans demonstrate slow but progressive growth of pulmonary lesions. During follow‐up, the patient was noted to have developed a pulmonary lesion that threatened obstruction of an airway and lower lobe collapse. This was ablated using a bronchoscopic ND‐YAG (neodymium‐doped yttrium aluminum garnet) laser (Fig. 1e, f).

The second patient with pulmonary cylindromas was the proband's mother. She had undergone scalp excision and was an ex‐smoker. She was found to have four small pulmonary nodules that were detected on a chest radiograph undertaken as part of investigations for breathlessness at the age of 80. Following pulmonary function tests, her primary respiratory diagnosis was chronic obstructive airways disease, and the pulmonary cylindromas were considered not to be symptomatic because of their size and location. Serial CTs over a 2‐year period confirmed absence of growth of these lesions. This patient succumbed to bronchopneumonia, and pulmonary tumours were sampled at autopsy. A third patient aged 51 was the half‐sister of the proband, who did not have any pulmonary lesions despite also having a severe cutaneous phenotype.

Histopathology of pulmonary and cutaneous lesions were compared in both patients, revealing the pulmonary lesions shared histological features with cutaneous cylindroma. There were no features in the pulmonary lesions such as cytological atypia to suggest malignancy (Fig. 2c, d). Ki‐67, TRKB and TRKC staining was performed to characterize protein expression in skin and lung tumours, supporting similarities in both tissues (Fig. 2e–h).

**Figure 3:**
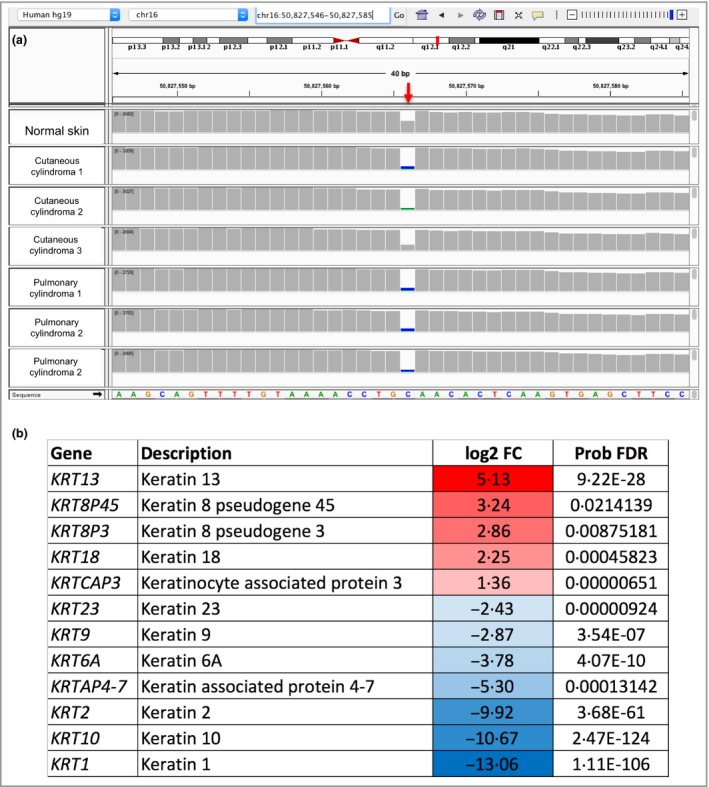
Pulmonary cylindromas demonstrate loss of heterozygosity and express a cytokeratin profile seen in cutaneous cylindroma. (a) Loss of heterozygosity at the *CYLD* locus in cutaneous and pulmonary cylindroma. The germline mutation (red arrow – c.2460delC) demonstrated in lung and skin tumours from this patient in relation to normal control (50% reads) and demonstrate 70–89% reads carrying the germline mutation, reflecting varying levels of infiltration of the tumour with inflammatory cells. (b) Cytokeratins differentially expressed in pulmonary cylindromas compared with perilesional lung tissue share similarity with those differentially expressed in cutaneous cylindroma. Transcriptomes of three samples of pulmonary cylindroma were compared with normal control skin of unaffected patients, and differentially expressed keratin and keratin‐related genes with a false discovery rate of <0·05 after correction for multiple hypothesis testing are shown. Genes that are increased in expression in pulmonary tumours compared with control skin are shown in red and genes that are reduced are shown in blue. BP, base pair; log2 FC, log^2^ fold change; Prob FDR, false discovery rate.

Using fresh pulmonary and skin tumour tissue, we characterized the *CYLD* locus using polymerase chain reaction coupled with next‐generation sequencing, demonstrating loss of heterozygosity, a feature reported in 75% of cutaneous cylindroma (Fig. 3a).^2^ Transcriptomic profiling was performed of lung tumours. We focused on cytokeratin profiles and compared these with an existing dataset of differentially expressed keratins in inherited CYLD defective tumours from a cohort of 32 tumours (Table S1; see Supporting Information).^2^ Lung tumours demonstrated a similar transcriptomic cytokeratin profile (including increased expression of *KRT13*,* KRT18* and *KRTCAP3*) to cutaneous cylindroma (Fig. 3b; Table S1), in addition to other markers in cutaneous cylindroma such as increased *NTRK2* (TRKB) and *NTRK3* (TRKC) expression (data not shown).

## Discussion

Here we present data that extends the phenotype seen in *CYLD* mutation carriers to include multiple pulmonary cylindromas. We reviewed all historic cases of patients with features suggesting CCS and pulmonary tumours of which there were four (Table 2). Three had features suggesting dissemination of a malignant cutaneous cylindroma, including an infiltrative growth pattern in the lung, nodal disease or liver or bone metastases. Only one case shared ‘benign’ features similar to our patients. Vernon *et al*.^7^ report the occurrence of a 46‐year‐old female patient with a severe phenotype of CCS and a solitary benign lung cylindroma discovered on autopsy following myocardial infarction.^7^ Histology of pulmonary and cutaneous cylindromas was reported as identical. The two cases of our female patients with the pathogenic c.2460delC mutation^6^, ^8^ add to the understanding of this uncharacterized aspect of CCS, as unlike the report by Vernon *et al*., they demonstrate for the first time that multiple pulmonary lesions may present with symptoms. We also demonstrate that two additional cases of CCS (one individual with the same mutation c.2460delC and another with c.1112C>A) do not have these tumours by opportunistically reviewing CT data that were available. This suggests that not all patients with germline *CYLD* mutations have radiologically detectable pulmonary lesions. Further work such as prospectively designed magnetic resonance imaging studies of the lungs of asymptomatic individuals is needed to estimate the true prevalence of this pulmonary phenotype in these cohorts.

**Table 2:** Cases of inherited cylindromas and pulmonary involvement

Author	Case details
Pingitore and Campani^16^	A 63‐year‐old man with a salivary gland tumour and multiple scalp cylindromas. Some of these were ulcerated and bleeding and malignancy was not excluded. Noted to have both hilar lymph node and lung metastases that showed cylindroma architecture histologically
Vernon *et al*.^7^	A 46‐year‐old woman with multiple cylindromas and a solitary benign lung cylindroma. The histology of the single pulmonary and cutaneous cylindroma were reported as being identical. No lymphadenopathy detected, or other features of metastatic disease at postmortem
Gerretsen *et al*.^17^	A review of 28 cases of malignant cutaneous cylindromas. In four of these cases there was insufficient information available to analyse the data. Malignant transformation was found in nine patients (37·5%) with a solitary cutaneous cylindroma and 15 patients (62·5%) with multiple cutaneous cylindromas. In 11 patients (46%), metastases were found, most often in lymph nodes, liver and skeletal spine, but also in lung, rib, femur, stomach and thyroid – 6 of 11 patients had a solitary cylindroma and 5 of 11 patients had multiple cylindromas
Volter *et al*.^18^	A 55‐year‐old woman with a history of multiple cylindromas, 35 years following diagnosis presented with a large right occipital scalp tumour with intracranial invasion; staging revealed multiple metastases in both lungs. The patient died 1 month later

Two mechanisms may account for the development of pulmonary tumours seen in our patients where there is an absence of nodal disease or a history of malignant cylindroma. One is that the epithelial cells in the lung may be an unrecognized site in these patients susceptible to tumour formation, and that the tumours arising here may be associated with smoking‐induced mutations. This is not supported by the cytokeratin profile, which does not demonstrate lung cytokeratins such as 5, 7 and 19.^9^ The alternate possibility, which is favoured, is that cutaneous cylindroma cells metastasize from apparently benign cylindroma and seed in the lung. The concept of primary tumours that appear benign from a clinical and pathological perspective yet metastasize is long recognized but controversial.^10^ Transcriptomic profiling of pulmonary tumours in our case demonstrated a cytokeratin profile consistent with cutaneous cylindroma, a finding which does not prove metastasis, but is supportive.

Cylindromas are thought to arise from the hair follicle,^11^ and it is of interest that a related appendageal tumour, hidradenoma, has also been thought to spread in this manner.^12^ It may be that these tumours share commonality in methods of dissemination. In our two patients, it is interesting to note that none of the prior excised tumours demonstrated malignant features on histology during 30 years of surgical care at our centre, nor did skin examination reveal clinically malignant tumours at presentation. The elusive ‘tumour of origin’ in the skin may, hence, not display distinguishing clinical features. Future whole genome studies of these tumours may reveal mutational signatures in these tumours, which will add further insights regarding the location of the cell of origin. Mutations in a tumour can now implicate ultraviolet radiation or smoking as carcinogens that played a role in tumour pathogenesis as these now have distinct recognizable mutational signatures in cancer.^13^, ^14^

Therapeutically, we report the successful use of bronchoscopic laser resection to maintain airway patency and prevent pulmonary collapse. Treatment of multiple lesions is more challenging. Dysregulated TRK signalling has been proposed as a therapeutic target in *CYLD* defective tumours.^2^ Expression of TRKs is seen in the pulmonary cylindromas, supporting the consideration of emerging oral TRK inhibitors in the management of these tumours.^15^ In summary, we describe the pulmonary phenotype of this poorly studied rare disease, describe monitoring and management of pulmonary cylindromas and add to the debate on benign metastases.

## Supporting information


**Fig S1.** A seven‐generation pedigree with *CYLD* cutaneous syndrome.

**Table S1** Differentially expressed cytokeratins and keratin related proteins seen in a dataset of 32 *CYLD* defective tumours compared with perilesional normal skin.

**Powerpoint S1** Journal Club Slide Set.

**Video S1** Author video.
