# Supplementary figures and images for: Inherited pulmonary cylindromas: extending the phenotype of CYLD mutation carriers
Source: Br J Dermatol. 2018 May 29;179(3):662–8. doi: 10.1111/bjd.16573 (PMC6175122; doi:10.1111/bjd.16573)

Supplementary Figure 1

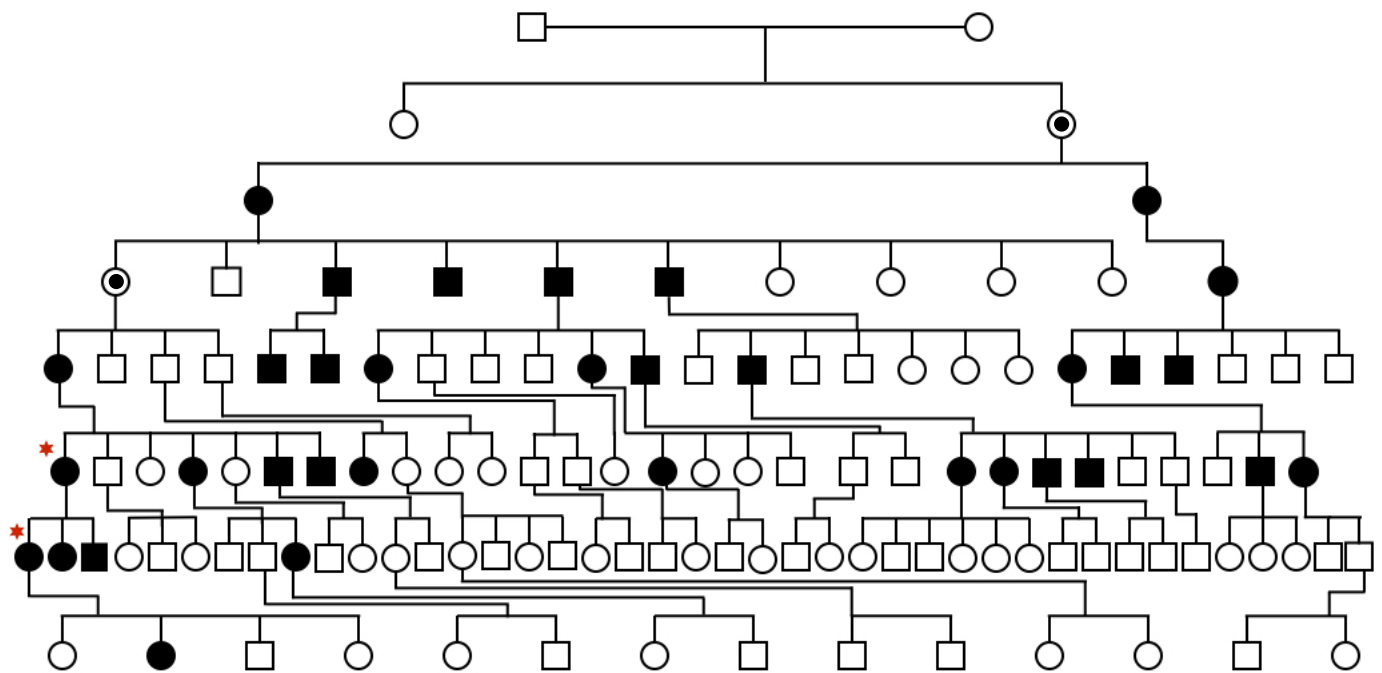

Supplement: Supplementary file 1 — Fig S1. A seven‐generation pedigree with CYLD cutaneous syndrome. [file BJD-179-662-s001.pdf]
