# Supplementary material for: Inherited pulmonary cylindromas: extending the phenotype of CYLD mutation carriers
Source: Br J Dermatol. 2018 May 29;179(3):662–8. doi: 10.1111/bjd.16573 (PMC6175122; doi:10.1111/bjd.16573)
Supplement: Supplementary file 2 — Table S1 Differentially expressed cytokeratins and keratin related proteins seen in a dataset of 32 CYLD defective tumours compared with perilesional normal skin. [file BJD-179-662-s002.docx]

**Supplementary Table S1.** Keratin and keratin related genes that are differentially expressed in a collection of 32 CYLD defective tumours compared to 10 control skin samples. Genes that are increased in expression in tumours compared to perilesional skin are shown in red and genes that are reduced are shown in blue.

| **Gene Symbol** | **Tumour.DiffScore** |
| --- | --- |
| *KRT8* | 114.5383 |
| *KRTCAP3* | 53.97984 |
| *KRT13* | 53.57121 |
| *KRT7* | 30.87368 |
| *KRT18* | 17.00012 |
| *KRT9* | -13.04201 |
| *KRTAP10-12* | -13.0817 |
| *KRTAP9-4* | -13.32384 |
| *KRTAP17-1* | -13.3525 |
| *KRTAP9-3* | -13.38516 |
| *KRTAP5-6* | -14.05755 |
| *KRTAP4-14* | -14.08497 |
| *KRT33A* | -14.33143 |
| *KRT6E* | -15.34284 |
| *KRTAP8-1* | -15.41039 |
| *KRTAP1-3* | -15.48424 |
| *KRT26* | -15.52445 |
| *KRTAP5-11* | -15.63616 |
| *KRTAP4-7* | -15.67754 |
| *KRTAP5-5* | -15.85459 |
| *KRTAP5-8* | -16.04793 |
| *KRTAP11-1* | -16.25328 |
| *KRT25D* | -16.88432 |
| *KRT25A* | -16.98588 |
| *KRTHB5* | -17.25786 |
| *KRT6A* | -17.81757 |
| *KRT25C* | -23.23366 |
| *KRT1B* | -23.37233 |
| *KRT23* | -25.44301 |
| *KRT2A* | -33.54452 |
| *KRT10* | -68.38415 |
| *KRT1* | -75.8578 |
