# Supplementary material for: Inherited pulmonary cylindromas: extending the phenotype of CYLD mutation carriers
Source: Br J Dermatol. 2018 May 29;179(3):662–8. doi: 10.1111/bjd.16573 (PMC6175122; doi:10.1111/bjd.16573)
Supplement: Supplementary file 3 — Powerpoint S1 Journal Club Slide Set. [file BJD-179-662-s003.pptx]

## Slide 1
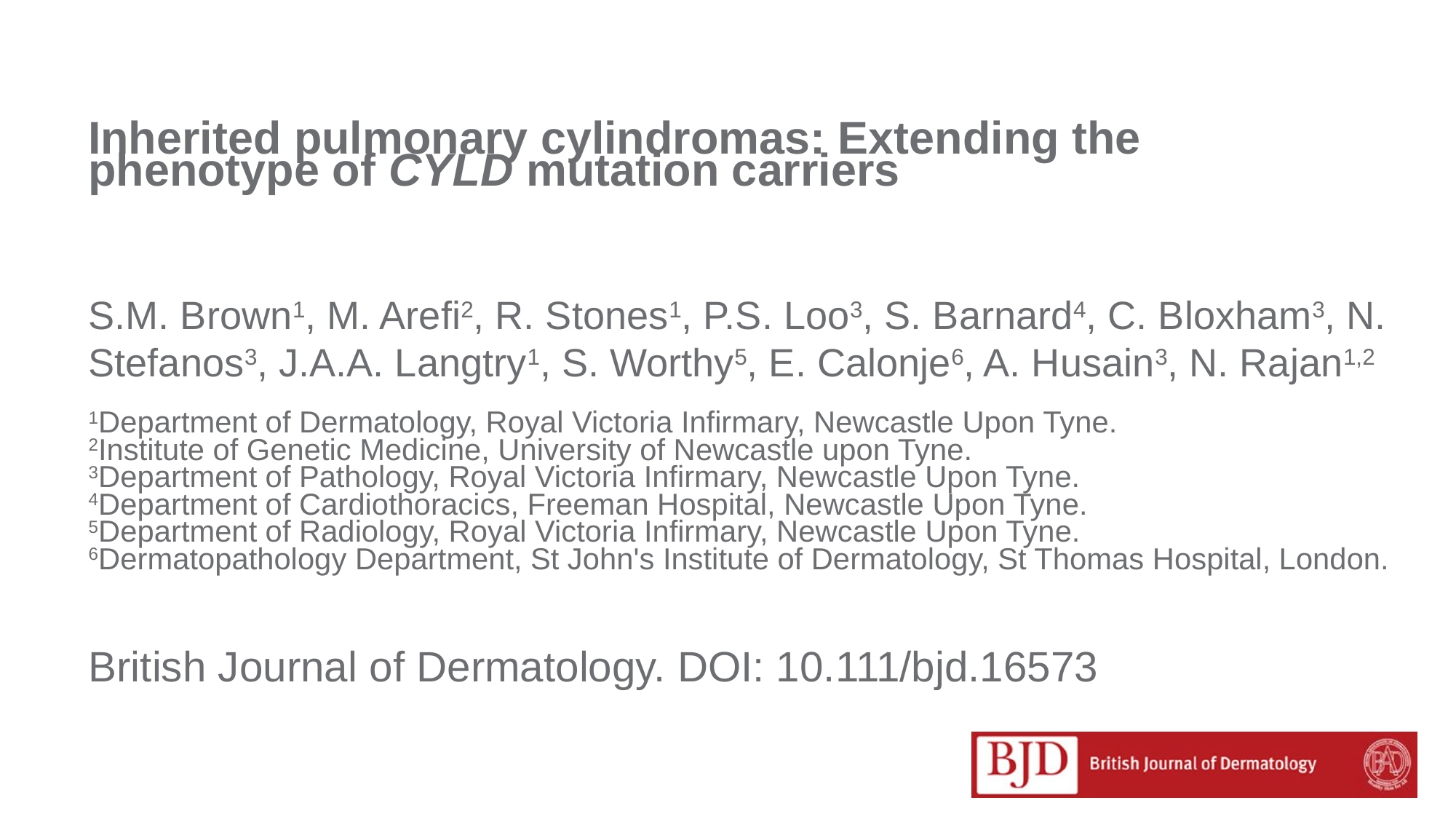

# Inherited pulmonary cylindromas: Extending the phenotype of CYLD mutation carriers
S.M. Brown1, M. Arefi2, R. Stones1, P.S. Loo3, S. Barnard4, C. Bloxham3, N. Stefanos3, J.A.A. Langtry1, S. Worthy5, E. Calonje6, A. Husain3, N. Rajan1,2
1Department of Dermatology, Royal Victoria Infirmary, Newcastle Upon Tyne.
2Institute of Genetic Medicine, University of Newcastle upon Tyne.
3Department of Pathology, Royal Victoria Infirmary, Newcastle Upon Tyne.
4Department of Cardiothoracics, Freeman Hospital, Newcastle Upon Tyne.
5Department of Radiology, Royal Victoria Infirmary, Newcastle Upon Tyne.
6Dermatopathology Department, St John's Institute of Dermatology, St Thomas Hospital, London.
British Journal of Dermatology. DOI: 10.111/bjd.16573

## Slide 2
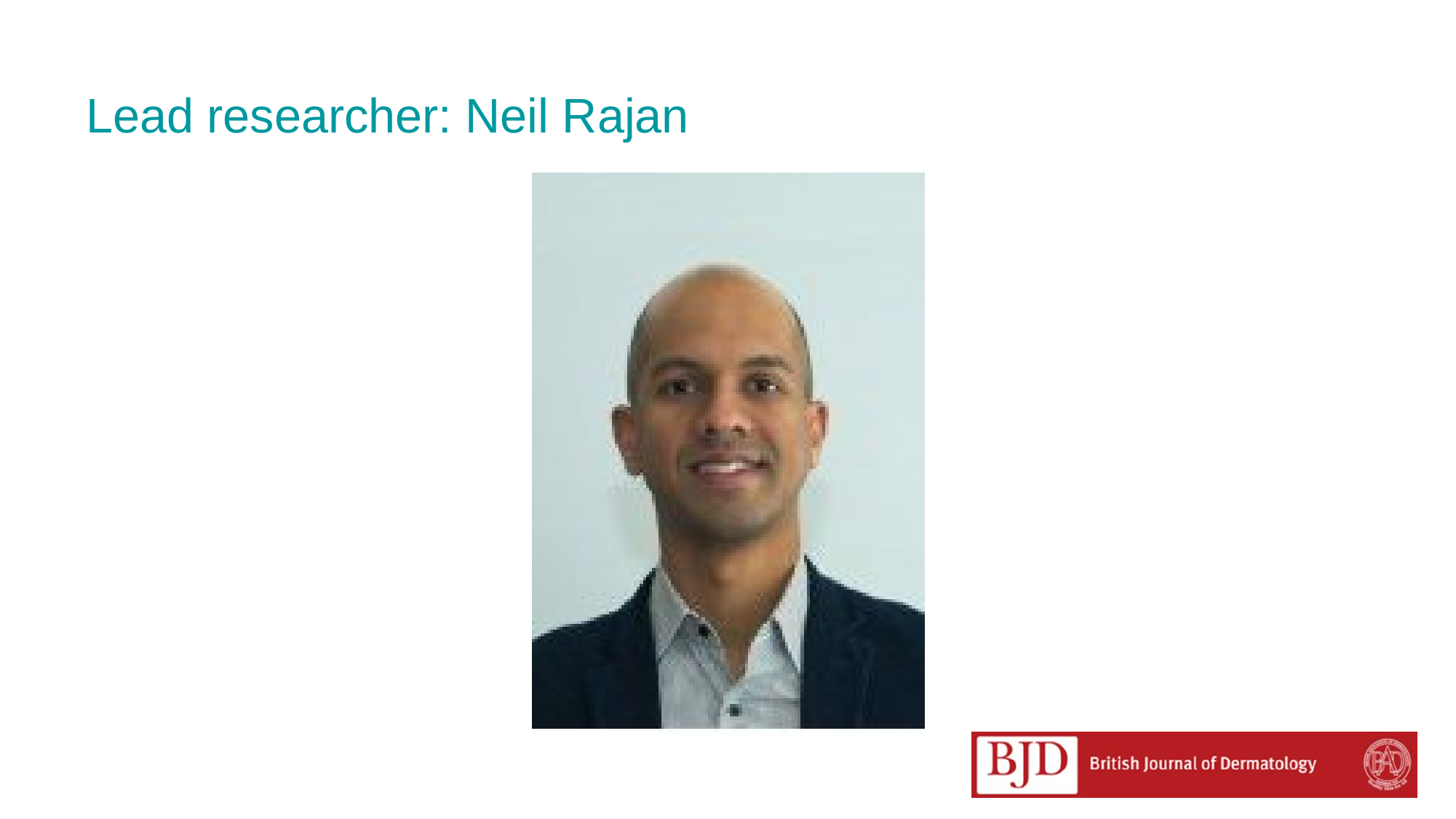

# Lead researcher: Neil Rajan

## Slide 3
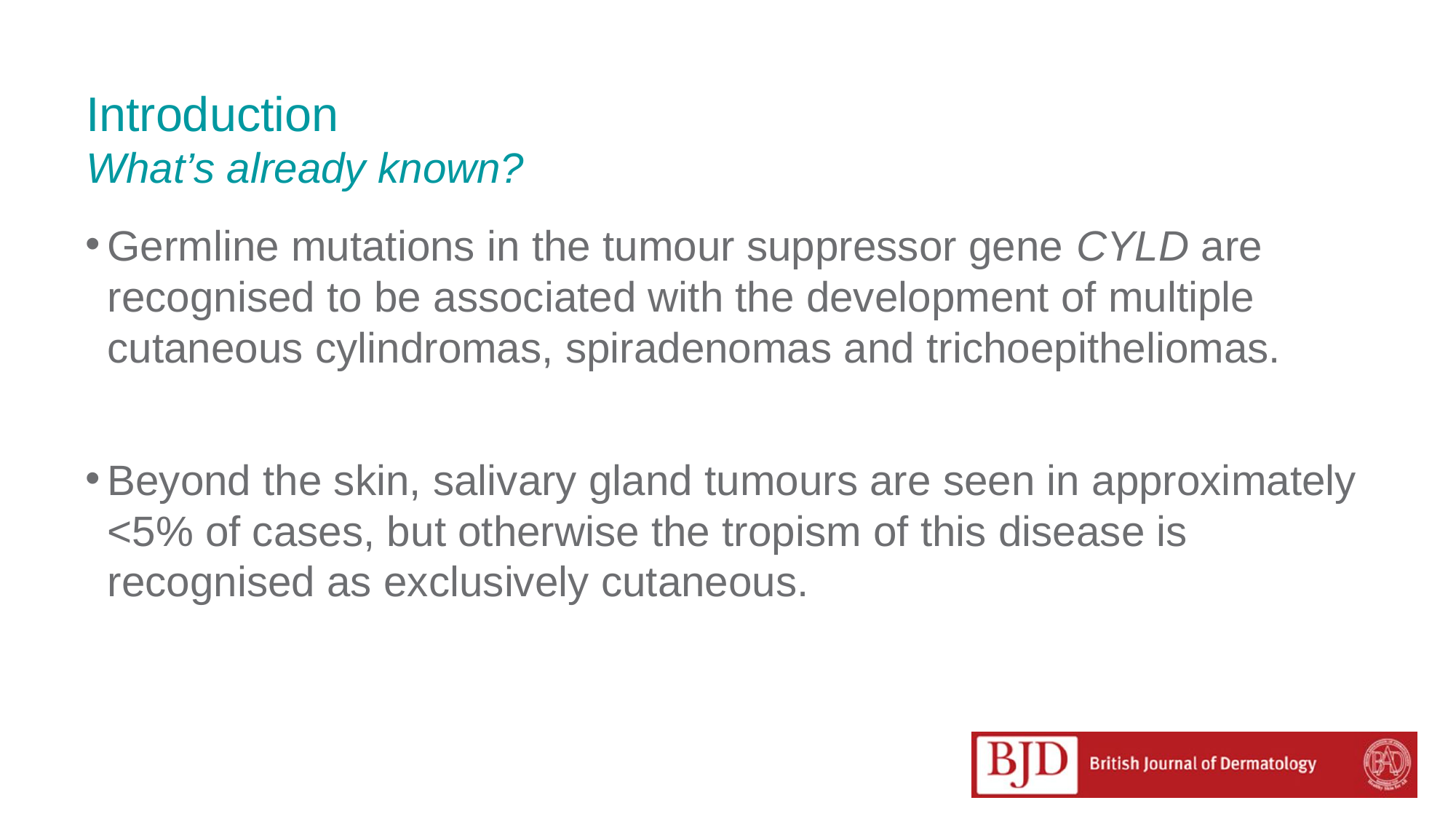

# Introduction What’s already known?
Germline mutations in the tumour suppressor gene CYLD are recognised to be associated with the development of multiple cutaneous cylindromas, spiradenomas and trichoepitheliomas.
Beyond the skin, salivary gland tumours are seen in approximately <5% of cases, but otherwise the tropism of this disease is recognised as exclusively cutaneous.

## Slide 4
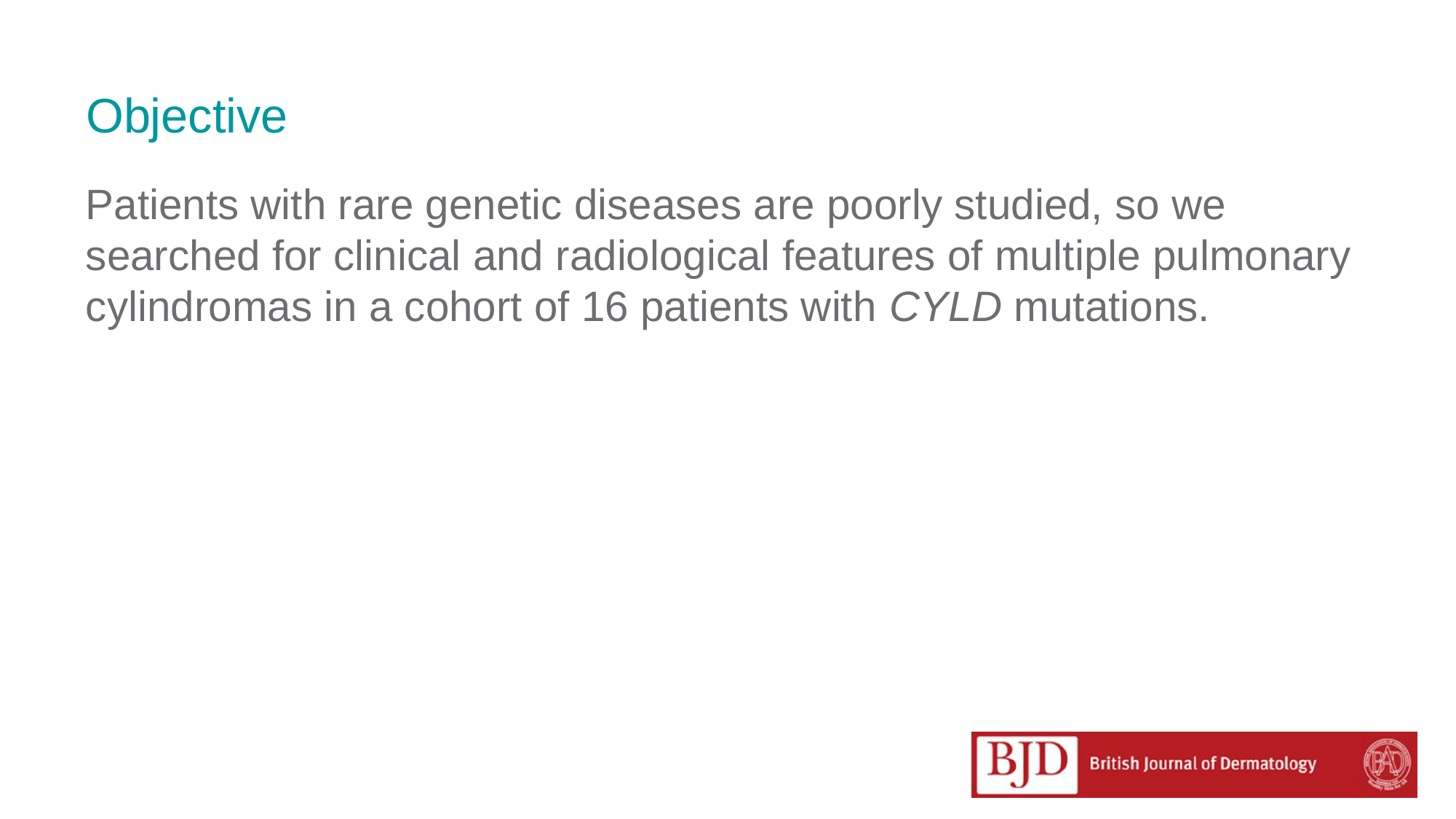

# Objective
Patients with rare genetic diseases are poorly studied, so we searched for clinical and radiological features of multiple pulmonary cylindromas in a cohort of 16 patients with CYLD mutations.

## Slide 5
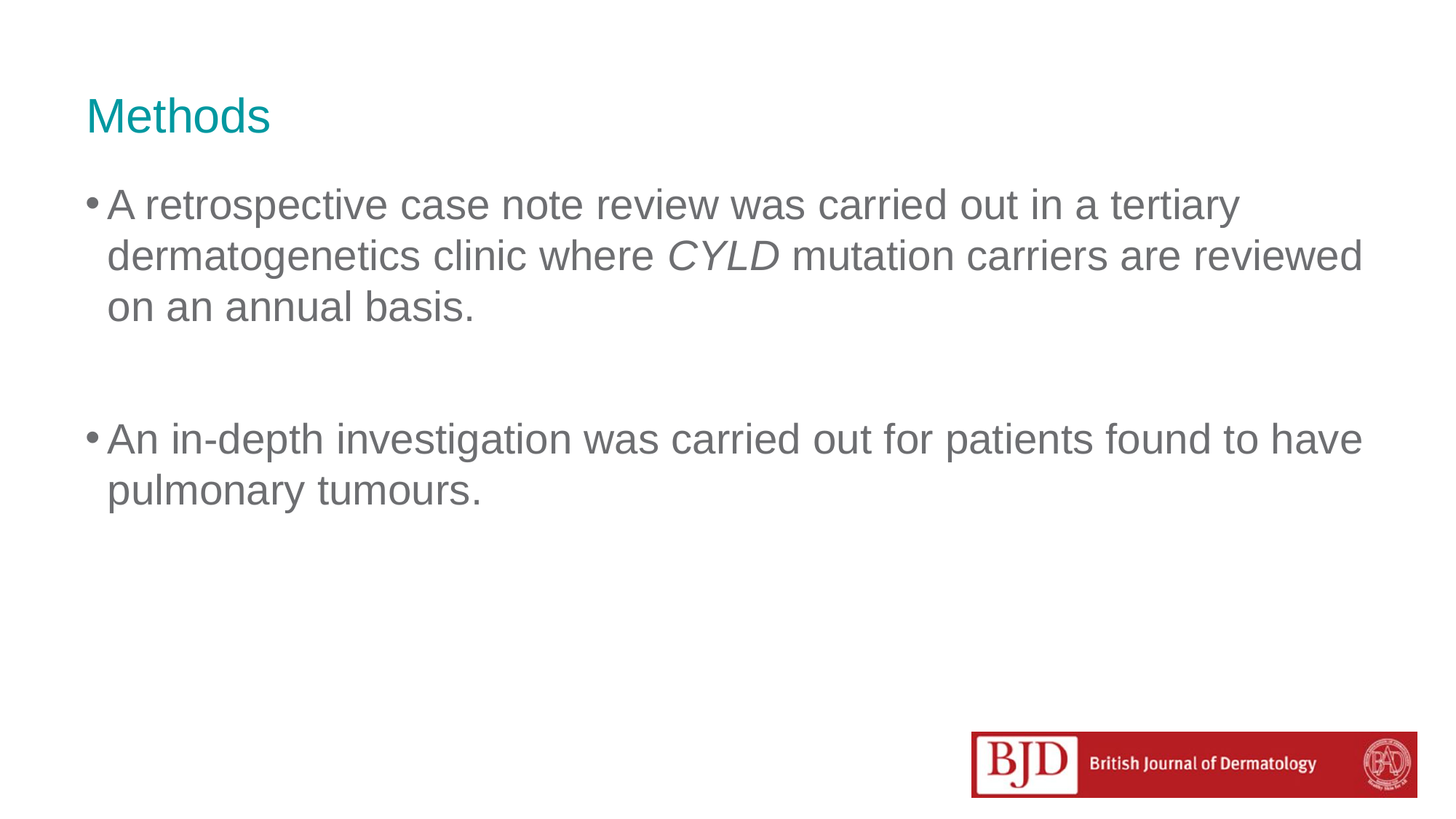

# Methods
A retrospective case note review was carried out in a tertiary dermatogenetics clinic where CYLD mutation carriers are reviewed on an annual basis.
An in-depth investigation was carried out for patients found to have pulmonary tumours.

## Slide 6
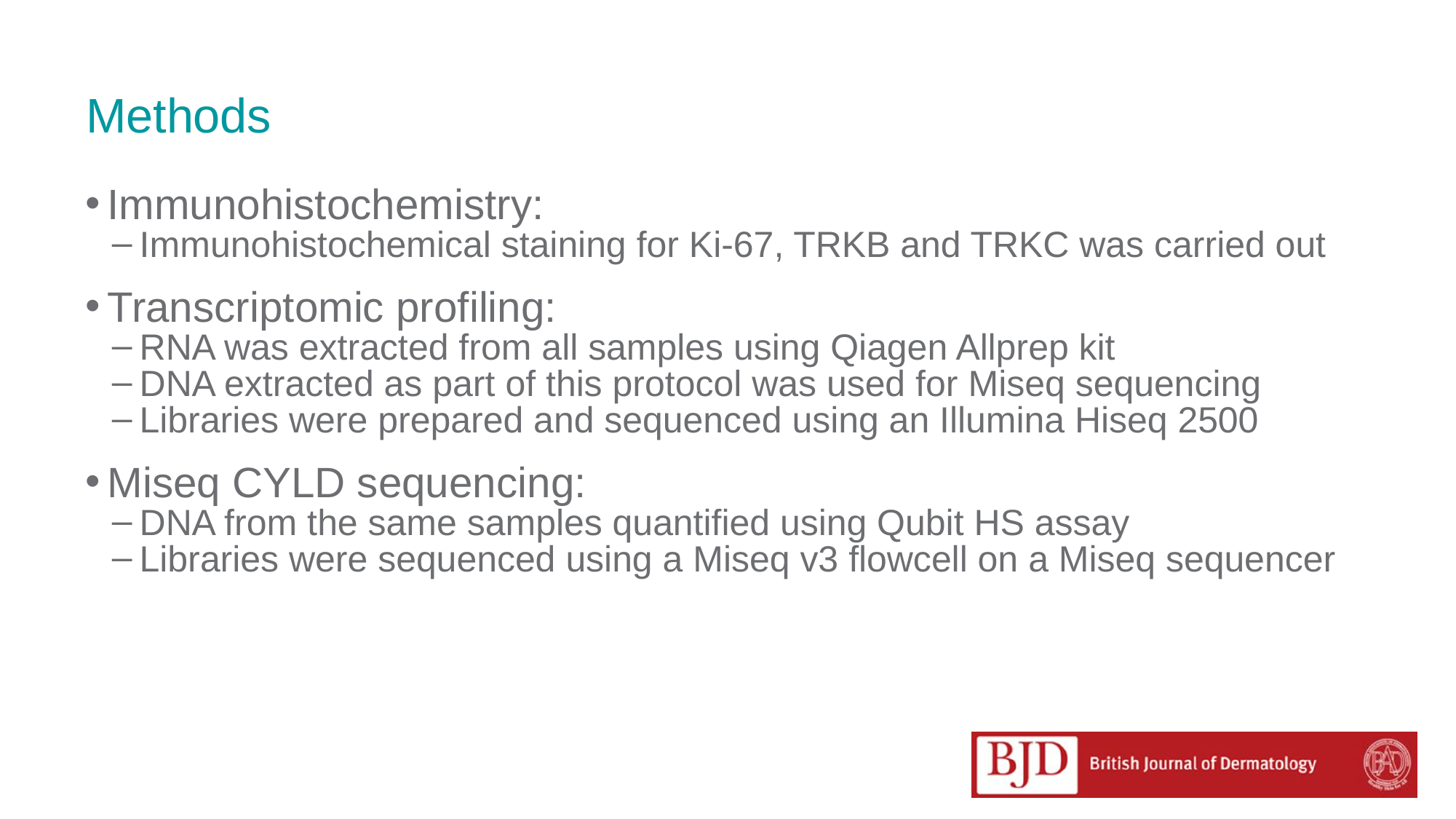

# Methods
Immunohistochemistry:
Immunohistochemical staining for Ki-67, TRKB and TRKC was carried out
Transcriptomic profiling:
RNA was extracted from all samples using Qiagen Allprep kit
DNA extracted as part of this protocol was used for Miseq sequencing
Libraries were prepared and sequenced using an Illumina Hiseq 2500
Miseq CYLD sequencing:
DNA from the same samples quantified using Qubit HS assay
Libraries were sequenced using a Miseq v3 flowcell on a Miseq sequencer

## Slide 7
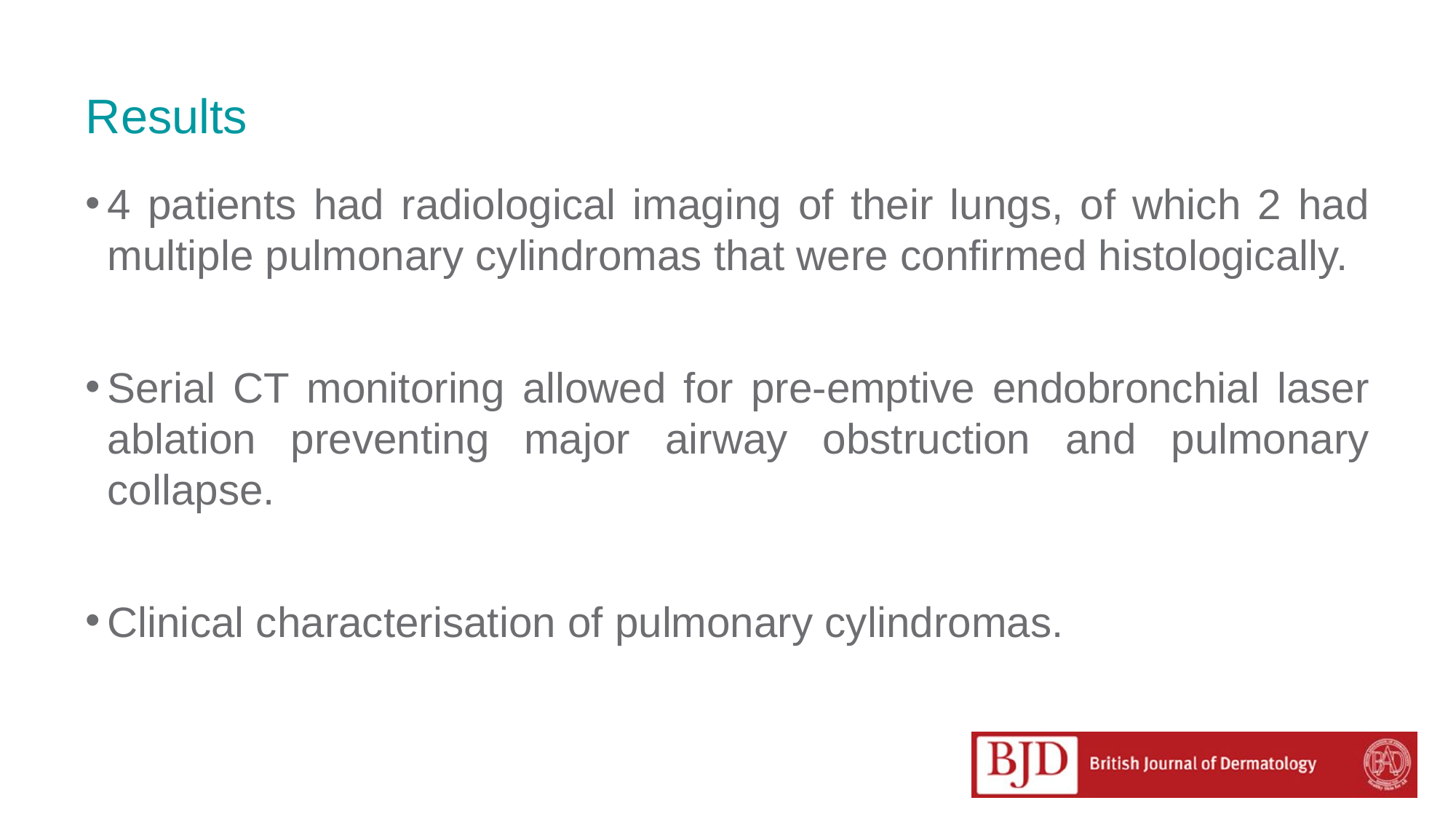

Results
4 patients had radiological imaging of their lungs, of which 2 had multiple pulmonary cylindromas that were confirmed histologically.
Serial CT monitoring allowed for pre-emptive endobronchial laser ablation preventing major airway obstruction and pulmonary collapse.
Clinical characterisation of pulmonary cylindromas.

## Slide 8
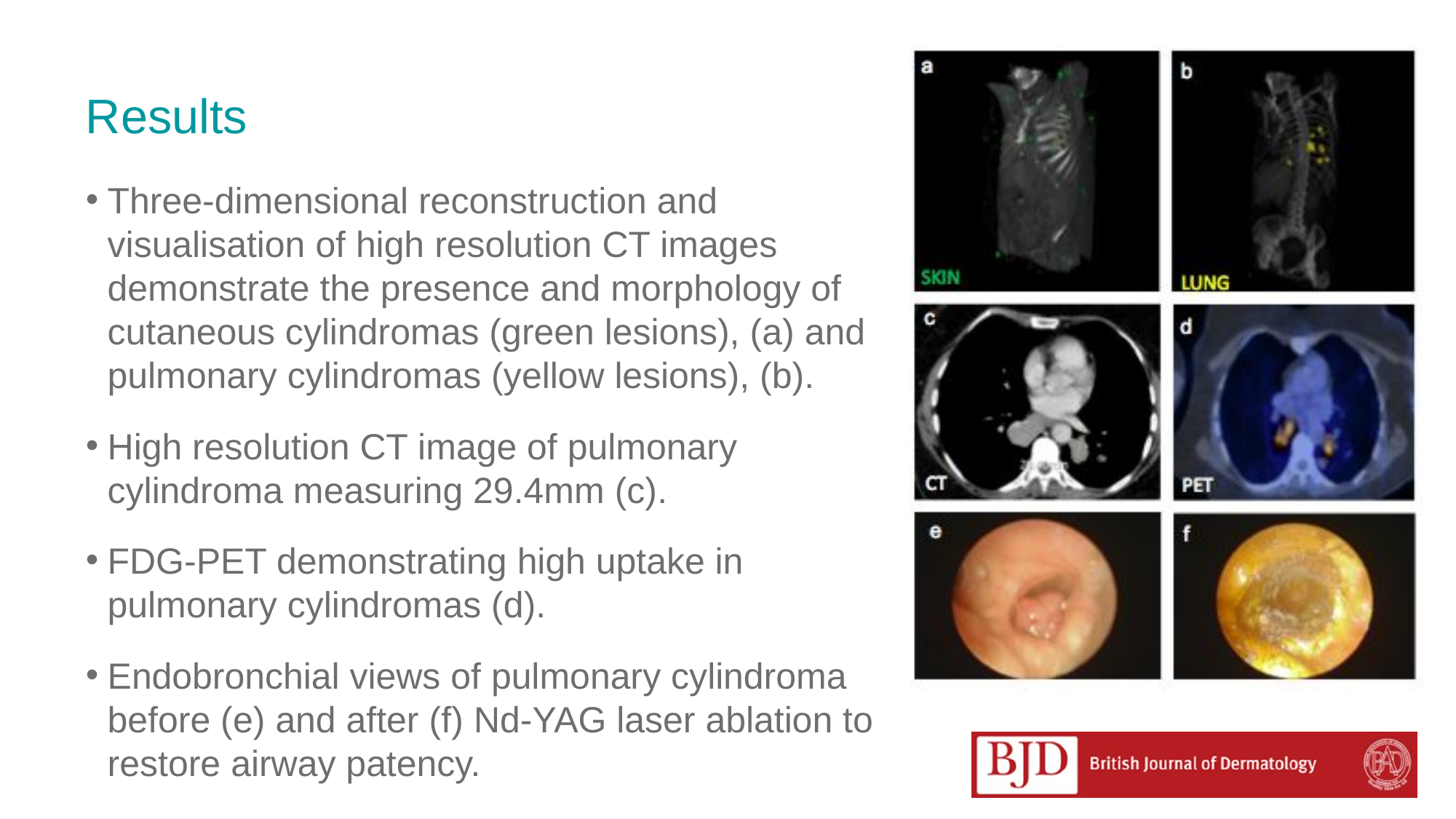

Results
Three-dimensional reconstruction and visualisation of high resolution CT images demonstrate the presence and morphology of cutaneous cylindromas (green lesions), (a) and pulmonary cylindromas (yellow lesions), (b).
High resolution CT image of pulmonary cylindroma measuring 29.4mm (c).
FDG-PET demonstrating high uptake in pulmonary cylindromas (d).
Endobronchial views of pulmonary cylindroma before (e) and after (f) Nd-YAG laser ablation to restore airway patency.

## Slide 9
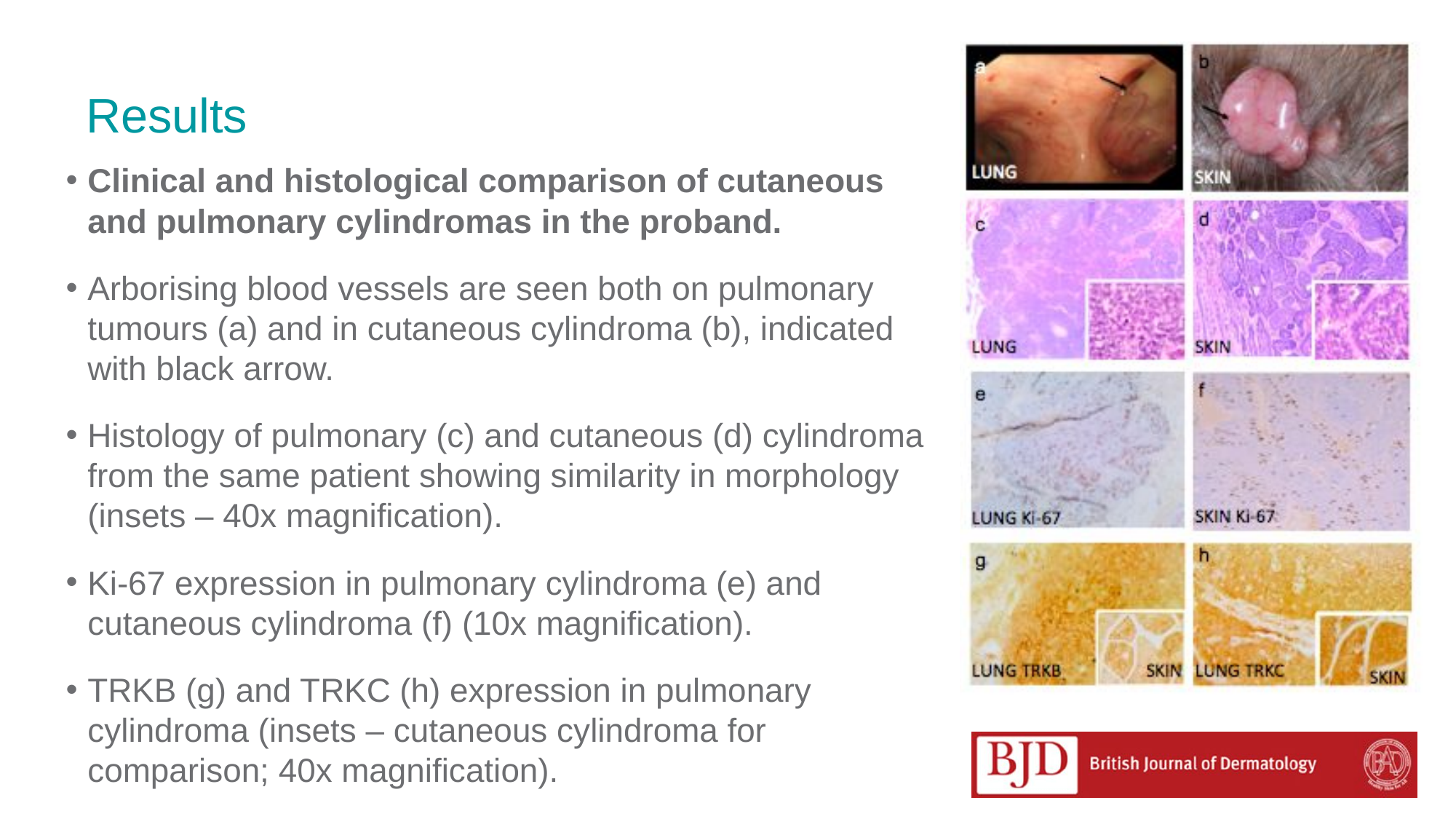

# Results
Clinical and histological comparison of cutaneous and pulmonary cylindromas in the proband.
Arborising blood vessels are seen both on pulmonary tumours (a) and in cutaneous cylindroma (b), indicated with black arrow.
Histology of pulmonary (c) and cutaneous (d) cylindroma from the same patient showing similarity in morphology (insets – 40x magnification).
Ki-67 expression in pulmonary cylindroma (e) and cutaneous cylindroma (f) (10x magnification).
TRKB (g) and TRKC (h) expression in pulmonary cylindroma (insets – cutaneous cylindroma for comparison; 40x magnification).

## Slide 10
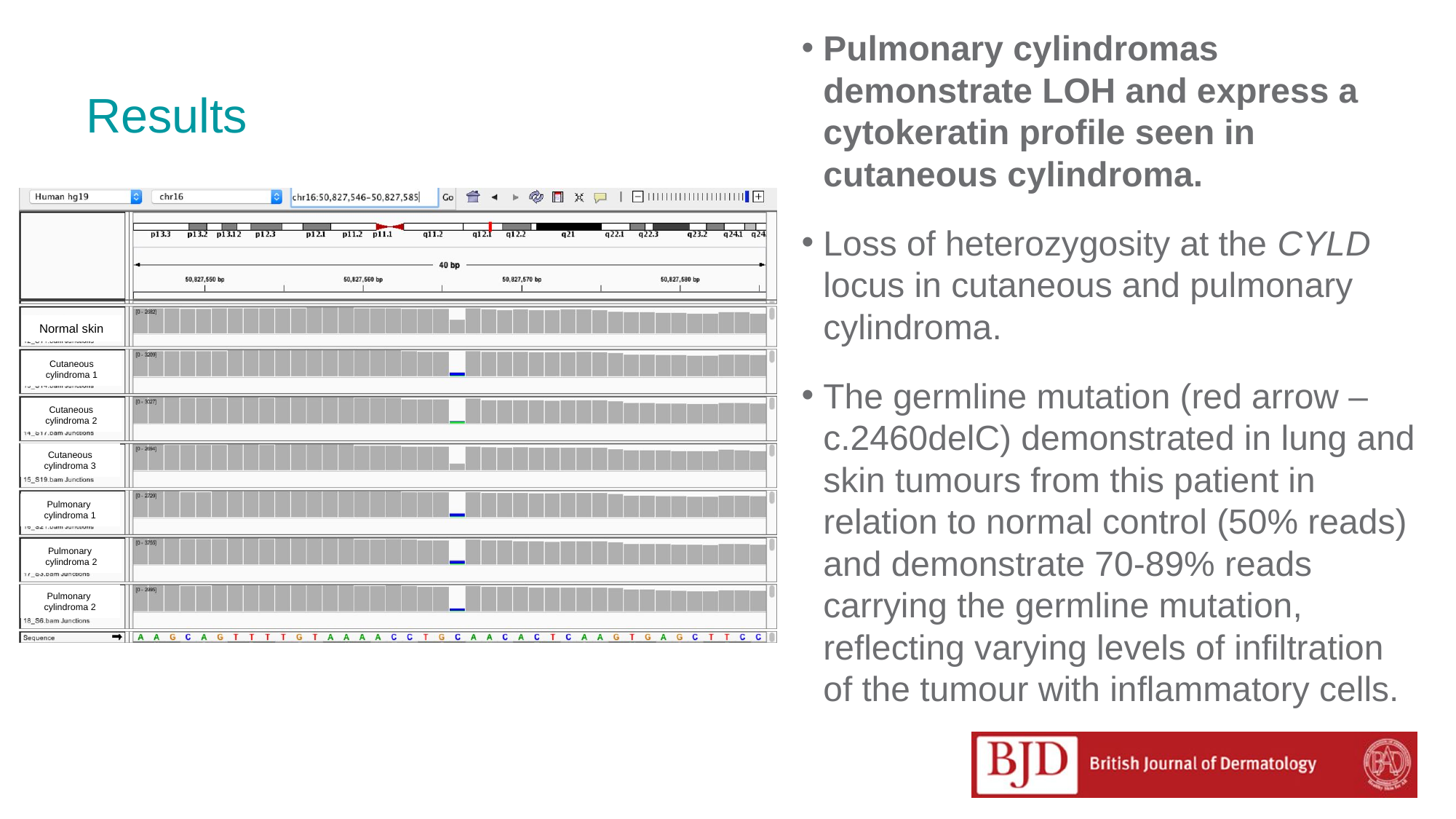

Pulmonary cylindromas demonstrate LOH and express a cytokeratin profile seen in cutaneous cylindroma.
Loss of heterozygosity at the CYLD locus in cutaneous and pulmonary cylindroma.
The germline mutation (red arrow – c.2460delC) demonstrated in lung and skin tumours from this patient in relation to normal control (50% reads) and demonstrate 70-89% reads carrying the germline mutation, reflecting varying levels of infiltration of the tumour with inflammatory cells.
# Results
Normal skin
Cutaneous cylindroma 1
Cutaneous cylindroma 2
Cutaneous cylindroma 3
Pulmonary
cylindroma 1
Pulmonary
cylindroma 2
Pulmonary
cylindroma 2

## Slide 11
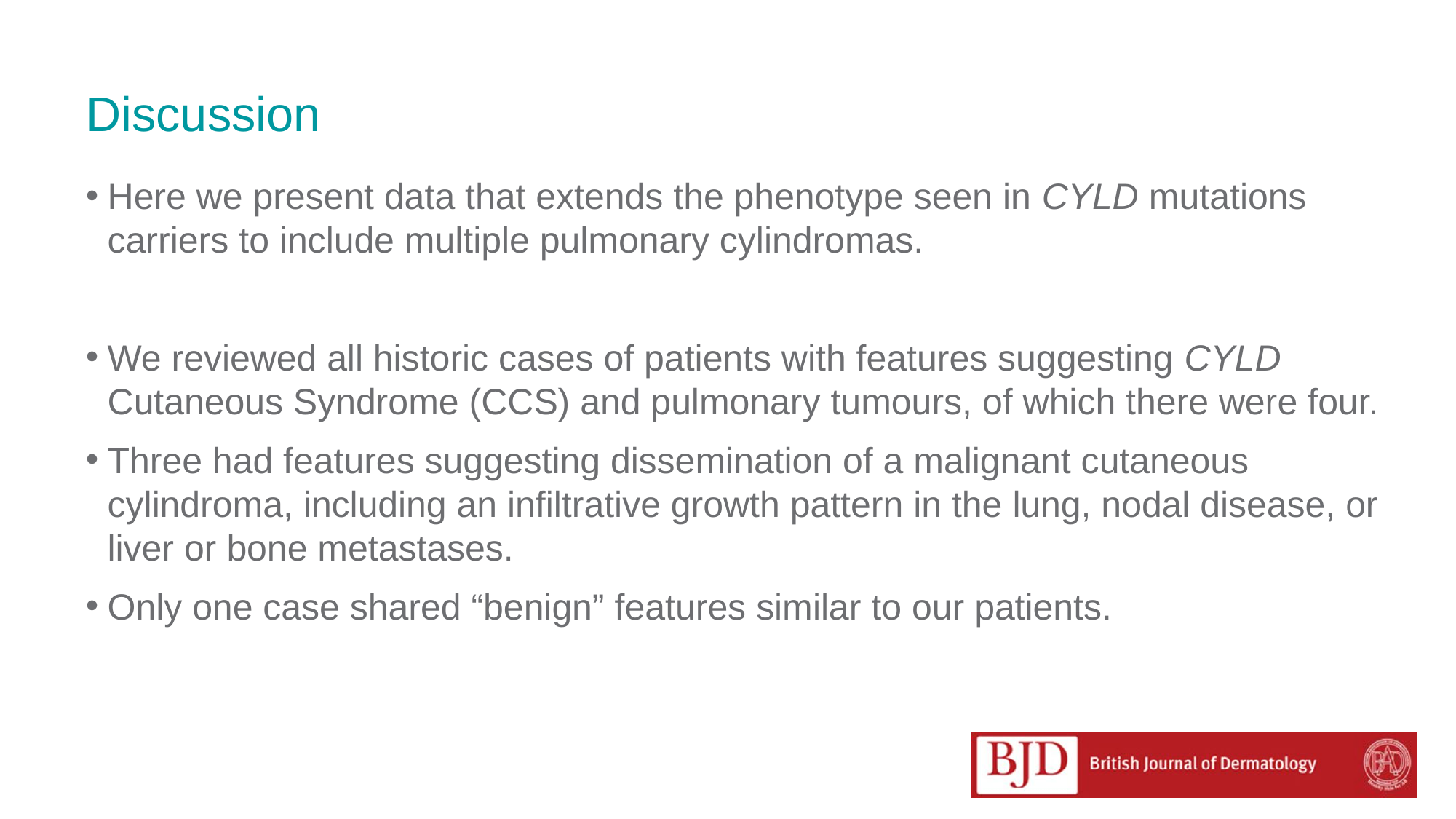

# Discussion
Here we present data that extends the phenotype seen in CYLD mutations carriers to include multiple pulmonary cylindromas.
We reviewed all historic cases of patients with features suggesting CYLD Cutaneous Syndrome (CCS) and pulmonary tumours, of which there were four.
Three had features suggesting dissemination of a malignant cutaneous cylindroma, including an infiltrative growth pattern in the lung, nodal disease, or liver or bone metastases.
Only one case shared “benign” features similar to our patients.

## Slide 12
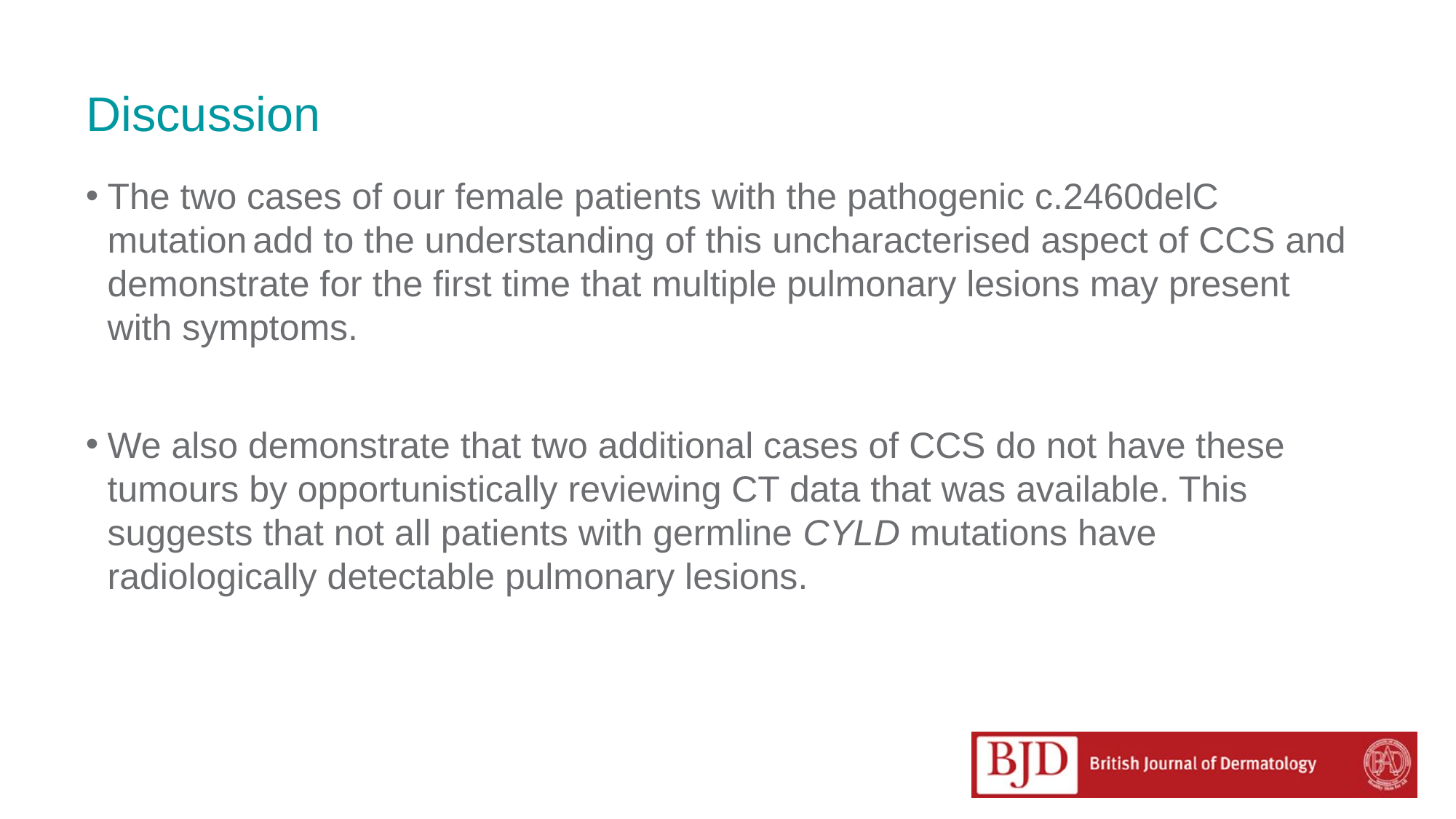

# Discussion
The two cases of our female patients with the pathogenic c.2460delC mutation add to the understanding of this uncharacterised aspect of CCS and demonstrate for the first time that multiple pulmonary lesions may present with symptoms.
We also demonstrate that two additional cases of CCS do not have these tumours by opportunistically reviewing CT data that was available. This suggests that not all patients with germline CYLD mutations have radiologically detectable pulmonary lesions.

## Slide 13
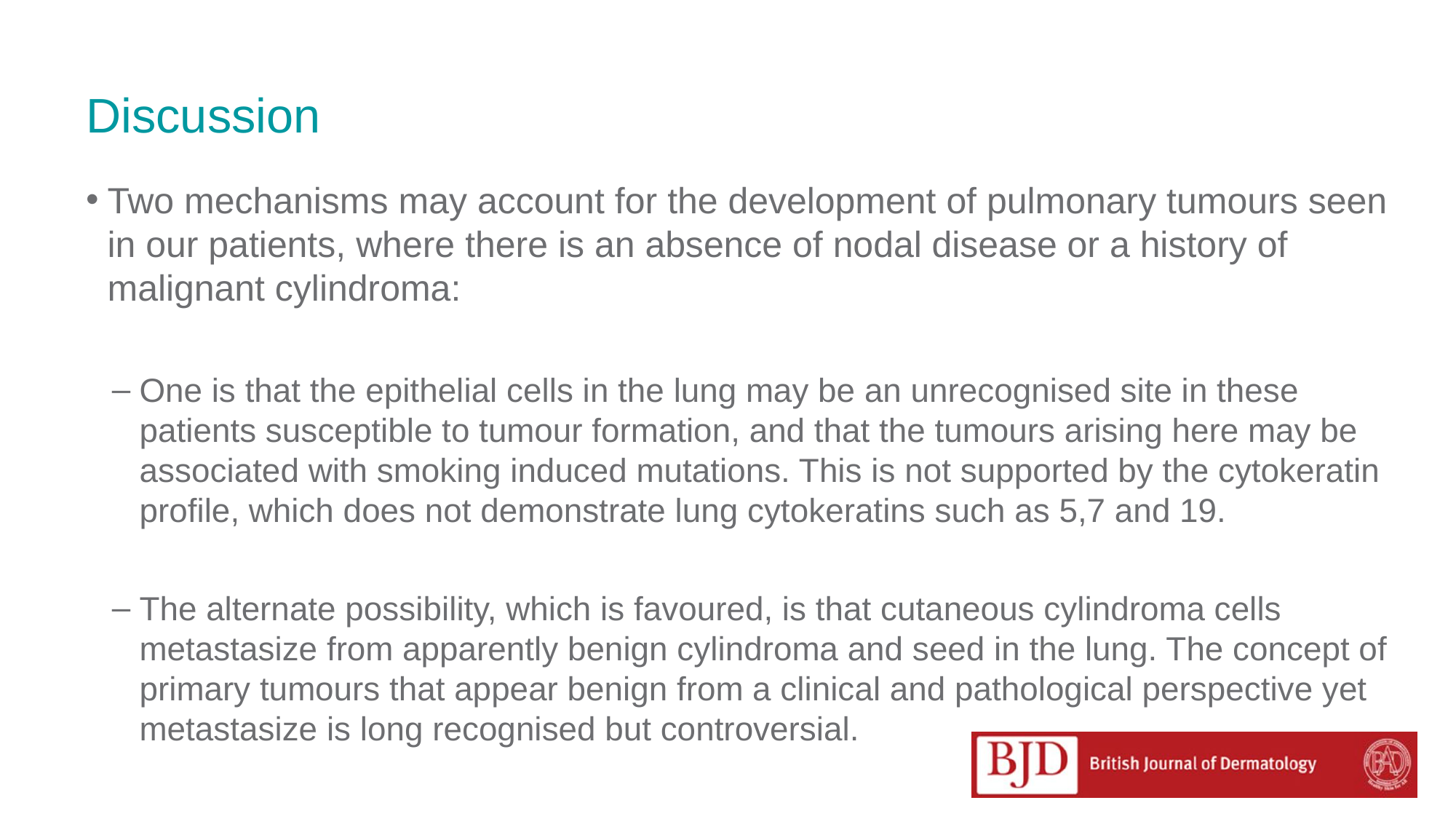

# Discussion
Two mechanisms may account for the development of pulmonary tumours seen in our patients, where there is an absence of nodal disease or a history of malignant cylindroma:
One is that the epithelial cells in the lung may be an unrecognised site in these patients susceptible to tumour formation, and that the tumours arising here may be associated with smoking induced mutations. This is not supported by the cytokeratin profile, which does not demonstrate lung cytokeratins such as 5,7 and 19.
The alternate possibility, which is favoured, is that cutaneous cylindroma cells metastasize from apparently benign cylindroma and seed in the lung. The concept of primary tumours that appear benign from a clinical and pathological perspective yet metastasize is long recognised but controversial.

## Slide 14
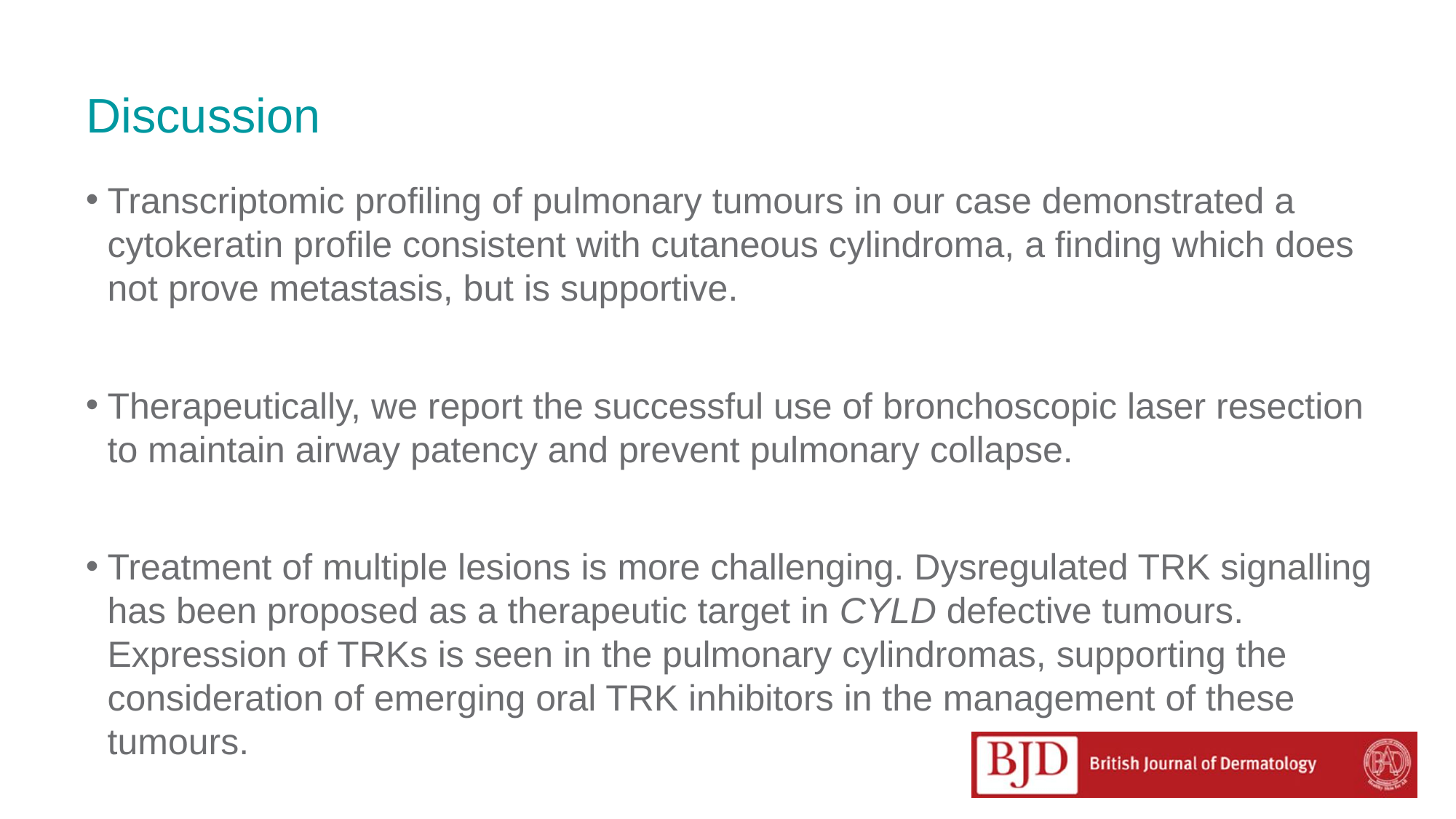

# Discussion
Transcriptomic profiling of pulmonary tumours in our case demonstrated a cytokeratin profile consistent with cutaneous cylindroma, a finding which does not prove metastasis, but is supportive.
Therapeutically, we report the successful use of bronchoscopic laser resection to maintain airway patency and prevent pulmonary collapse.
Treatment of multiple lesions is more challenging. Dysregulated TRK signalling has been proposed as a therapeutic target in CYLD defective tumours. Expression of TRKs is seen in the pulmonary cylindromas, supporting the consideration of emerging oral TRK inhibitors in the management of these tumours.

## Slide 15
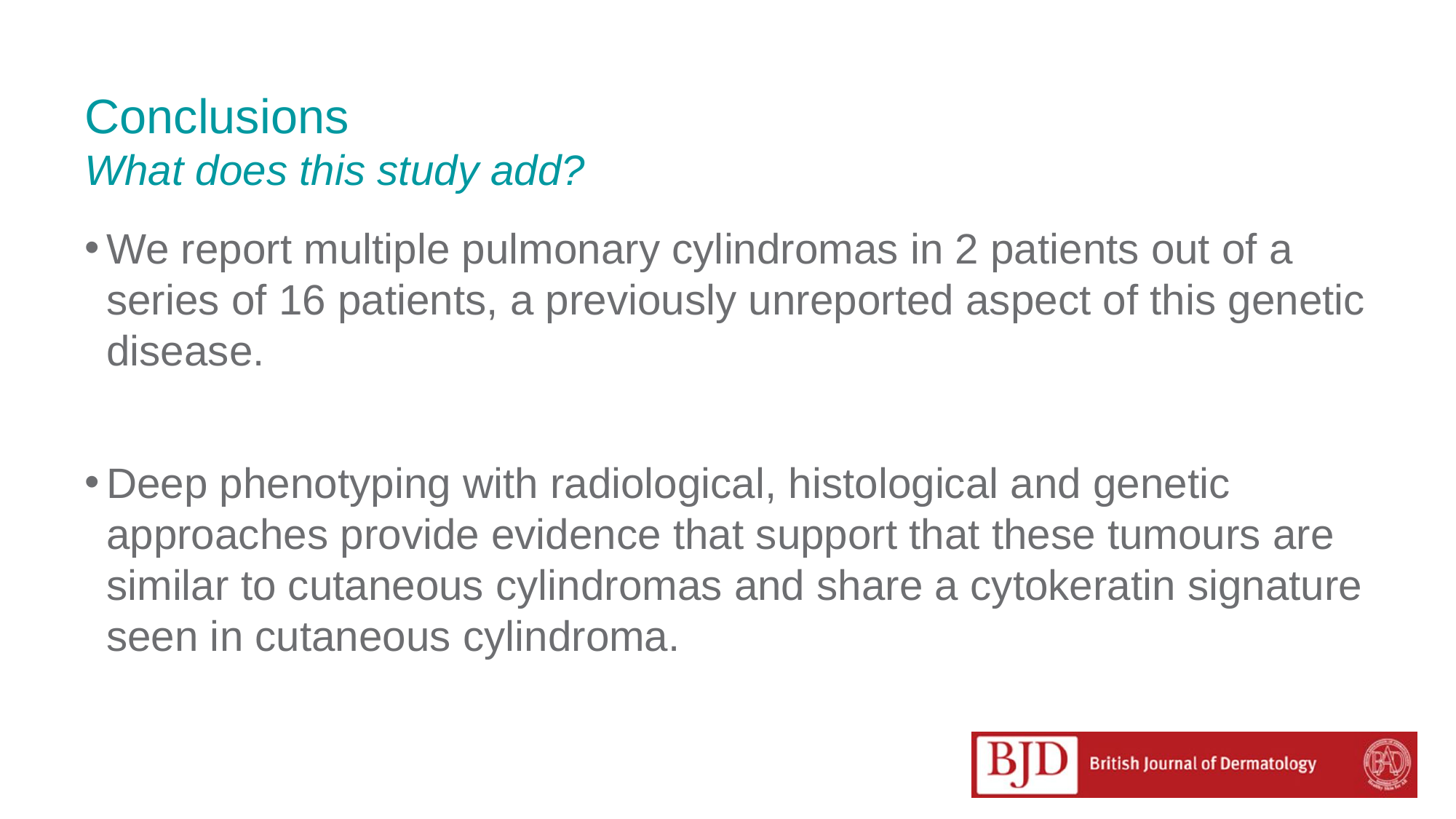

Conclusions
What does this study add?
We report multiple pulmonary cylindromas in 2 patients out of a series of 16 patients, a previously unreported aspect of this genetic disease.
Deep phenotyping with radiological, histological and genetic approaches provide evidence that support that these tumours are similar to cutaneous cylindromas and share a cytokeratin signature seen in cutaneous cylindroma.

## Slide 16
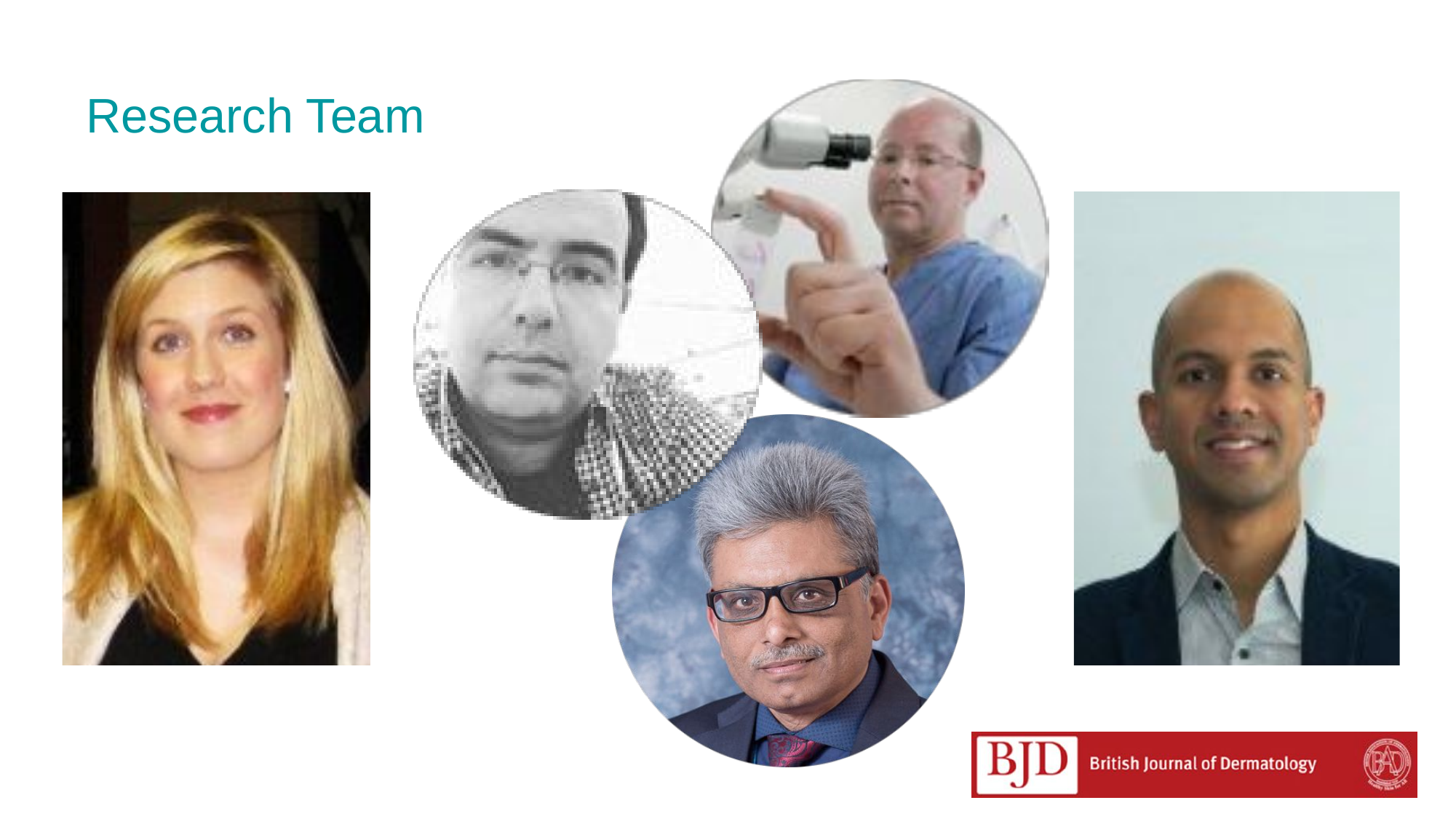

# Research Team

## Slide 17
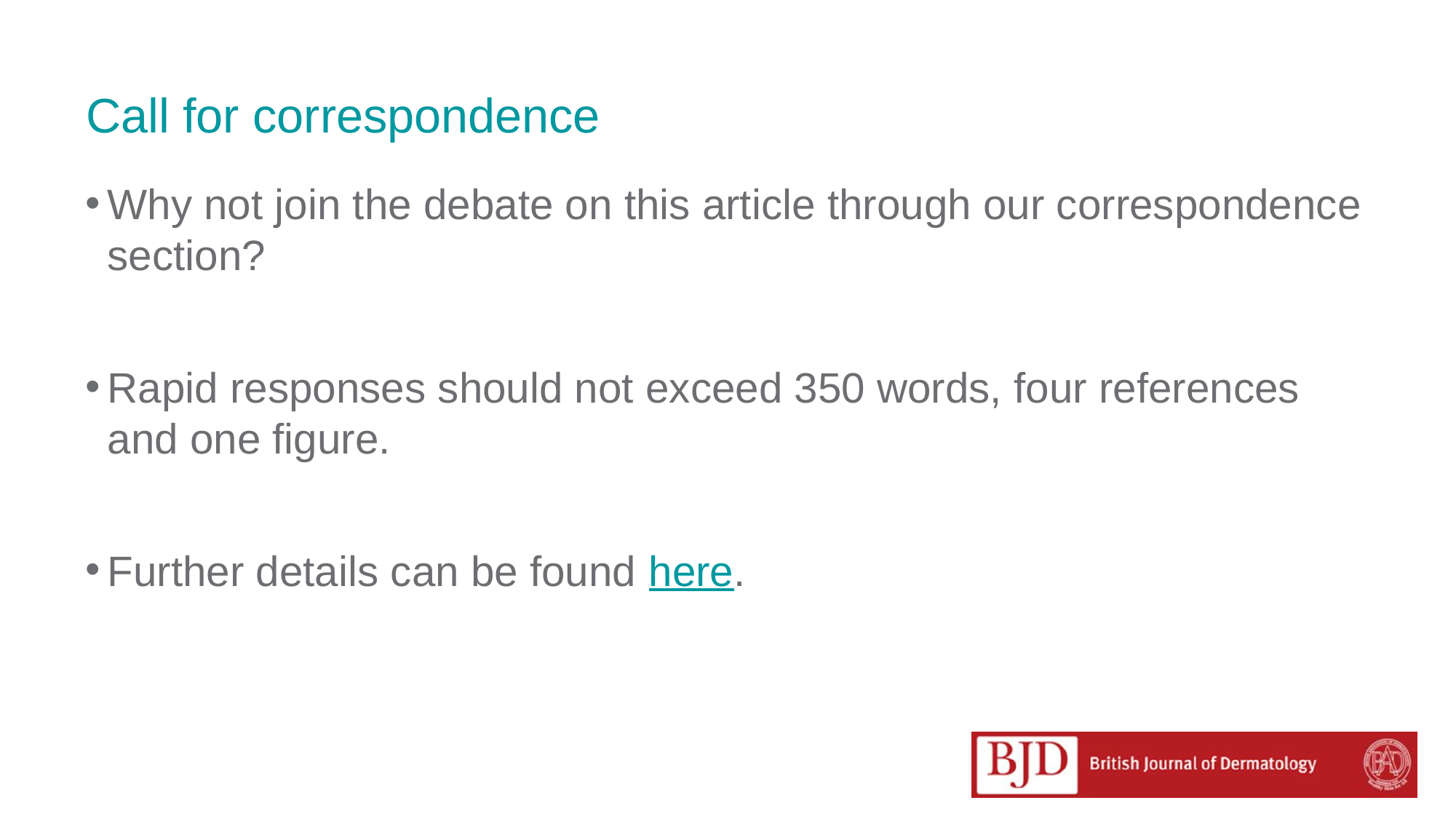

# Call for correspondence
Why not join the debate on this article through our correspondence section?
Rapid responses should not exceed 350 words, four references and one figure.
Further details can be found here.
